# Effectiveness of *R1-nj* Anthocyanin Marker in the Identification of In Vivo Induced Maize Haploid Embryos

**DOI:** 10.3390/plants12122314

**Published:** 2023-06-14

**Authors:** Luis Antonio Lopez, John Ochieng, Mario Pacheco, Leocadio Martinez, Hamilton Amoshe Omar, Manje Gowda, Boddupalli M. Prasanna, Kanwarpal S. Dhugga, Vijay Chaikam

**Affiliations:** 1 International Maize and Wheat Improvement Center (CIMMYT), Apdo. Postal 6-64106600, Mexico 06600, Mexico; 2 International Maize and Wheat Improvement Center (CIMMYT), ICRAF Campus, UN Avenue, Nairobi P.O. Box 1041-00621, Kenyam.gowda@cgiar.org (M.G.); b.m.prasanna@cgiar.org (B.M.P.)

**Keywords:** doubled haploids, *R1-nj* marker, embryo rescue, false positives, false detection rate, false negative rate

## Abstract

Doubled haploid (DH) technology has become integral to maize breeding programs to expedite inbred line development and increase the efficiency of breeding operations. Unlike many other plant species that use in vitro methods, DH production in maize uses a relatively simple and efficient in vivo haploid induction method. However, it takes two complete crop cycles for DH line generation, one for haploid induction and the other one for chromosome doubling and seed production. Rescuing in vivo induced haploid embryos has the potential to reduce the time for DH line development and improve the efficiency of DH line production. However, the identification of a few haploid embryos (~10%) resulting from an induction cross from the rest of the diploid embryos is a challenge. In this study, we demonstrated that an anthocyanin marker, namely *R1-nj*, which is integrated into most haploid inducers, can aid in distinguishing haploid and diploid embryos. Further, we tested conditions that enhance *R1-nj* anthocyanin marker expression in embryos and found that light and sucrose enhance anthocyanin expression, while phosphorous deprivation in the media had no affect. Validating the use of the *R1-nj* marker for haploid and diploid embryo identification using a gold standard classification based on visual differences among haploids and diploids for characteristics such as seedling vigor, erectness of leaves, tassel fertility, etc., indicated that the *R1-nj* marker could lead to significantly high false positives, necessitating the use of additional markers for increased accuracy and reliability of haploid embryo identification.

## 1. Introduction

The availability of an efficient and relatively simple in vivo protocol to develop completely homozygous inbred lines using doubled haploid (DH) technology greatly accelerated maize breeding in the last few decades. Simultaneous fixation of all segregating alleles into a homozygous condition in DH lines can be achieved in just two crop cycles, whereas seven crop cycles are required to obtain homozygosity at >99% of the loci from a segregating population in the conventional recurrent selfing method of inbred line development. Hundreds of thousands of genetically fixed inbred maize lines are now being routinely developed using DH technology by several multinational corporations every year, which is not feasible with conventional inbred line development methods. Genetically homozygous DH lines enable extraordinary selection efficiency from the very beginning of a breeding cycle in comparison to selecting plants/families that have different levels of homozygosity [1]. DH lines greatly enhance breeding efficiency by reducing time in inbred line development and simplifying breeding operations such as nursery management, inventory management, and seed shipments, thereby enabling cost savings [2,3].

Current DH production protocols in maize [4,5,6] involve crossing the source (donor) germplasm from which inbred lines are desired as a female parent with a maternal haploid inducer as a pollen parent. Inducers are generally equipped with dominantly expressed morphological or biochemical markers such as *R1-nj*, red root, or high oil that express phenotype at the seed or seedling stage and facilitate early identification of haploids from diploids. Doubling the haploid genome using mitosis-inhibiting chemicals enables the recovery of male and female fertility, thereby resulting in seed production for DH lines upon self-fertilization. Using modern haploid inducers, haploids can be obtained at a frequency of 8–15% [3,7]. Haploid identification using a single maker such as *R1-nj* is not so reliable because of the inhibition of marker expression and high rates of misclassification [8]; hence, inducers with multiple markers such as *R1-nj* and red root or *R1-nj* and high oil were developed to enhance the reliability of haploid identification [9,10]. Overall chromosomal doubling efficiencies using anti-mitotic chemicals were reported to be generally in the range of 5–35% [11,12,13]. Even though the current in vivo haploid induction-based DH production protocols are reliable for large-scale production of DH lines, there are further opportunities for increasing the efficiency of DH line production protocols that can result in reduced cost and faster delivery of DH lines.

Immature embryo rescue methods offer two distinct advantages over the currently used DH line development protocols. First, as embryos can be extracted from the ears pollinated with inducer pollen very early, such as 10–25 days after pollination, it can reduce the time for DH line production by up to six weeks [14]. Second, it can possibly increase the chromosomal doubling efficiency. Commonly used chromosomal doubling protocols involve treating 3–15-day old seedlings with anti-mitotic chemicals [11,12,13,15]. However, shoot apical meristem is deeply embedded in the shoot tissues and, hence, is difficult to access, limiting the efficiency of chromosomal doubling treatments.

Embryo rescue procedures for DH line production in maize typically involve isolating the embryos from the ears of the source populations that are crossed with a maternal haploid inducer, differentiating the haploid embryos from diploid embryos, subjecting the haploid embryos to chromosomal doubling treatments, generating and recovering the seedlings from the embryos, and seed production from doubled haploid plants. Embryo rescue offers easy access to all the meristematic cells in the developing embryo, which eventually give rise to all the plant parts, including reproductive tissues, making it possible to achieve maximum chromosomal doubling efficiency. Publications on maize embryo rescue protocols for DH line production are scarce other than proprietary protocols mentioned in patents [16,17]. A major step in DH line development is to effectively identify haploid embryos that occur infrequently compared with diploid embryos. Inducers with transgenic markers such as green fluorescent protein (GFP) [18], double fluorescent proteins (eGFP and dsRED) [19], RUBY reporter [20] and over-expression of ZmC1 and ZmR2 [21] were proposed to assist in haploid embryo identification. The objectives of this study were to: (1) assess if immature haploid and diploid embryos can be effectively separated based on the expression of *R1-nj* anthocyanin marker; (2) identify the conditions that enhance *R1-nj* anthocyanin marker expression; and (3) validate the haploid embryo identification based on a gold standard classification and molecular markers.

## 2. Results

### 2.1. R1-nj Anthocyanin Marker Can Aid in Distinguishing Haploid Embryos from the Diploid

**Figure 1:**
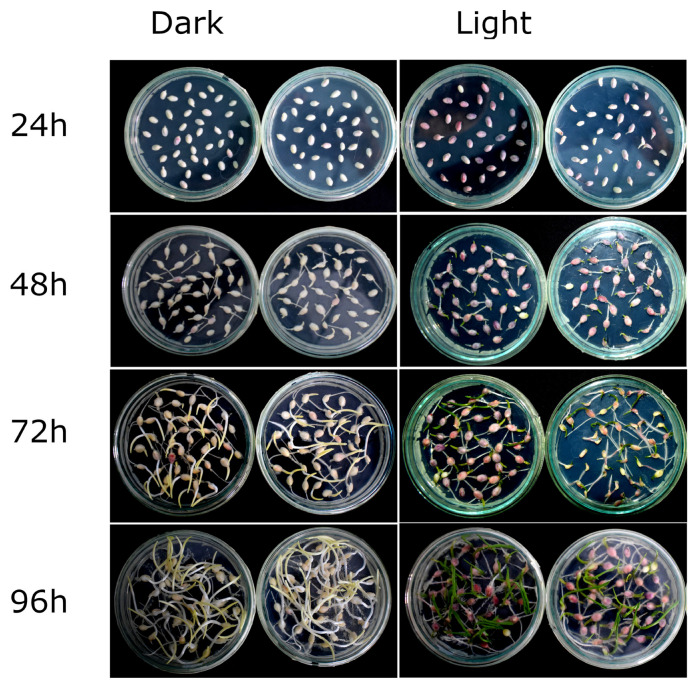
Effect of light on *R1-nj* marker expression in embryos incubated on MS-RD medium for 24, 48, 72, and 96 h.

When embryos were incubated on MS-RD medium in the dark, the expression of the *R1-nj* marker was completely lacking after 24 h, while it was faintly expressed in very few embryos after 48 h (Figure 1). The majority of the embryos did not express the anthocyanin marker even after 96 h of incubation on MS-RD medium in the dark. In contrast, embryos exposed to continuous light showed anthocyanin expression in 24 h and the intensity of anthocyanin coloration increased over time (Figure 1). It was interesting to note that some radicles also showed anthocyanin expression under continuous light, even though the expression was weak. This experiment established that the *R1-nj* anthocyanin marker could be expressed under certain conditions and could be used to distinguish the haploid and diploid embryos. However, it would be ideal to have a more intense expression of anthocyanins as early as possible so that chromosomal doubling involving expensive and toxic anti-mitotic chemicals can be targeted on fewer selected haploids. Hence, we evaluated conditions that can enhance anthocyanin expression.

**Figure 2:**
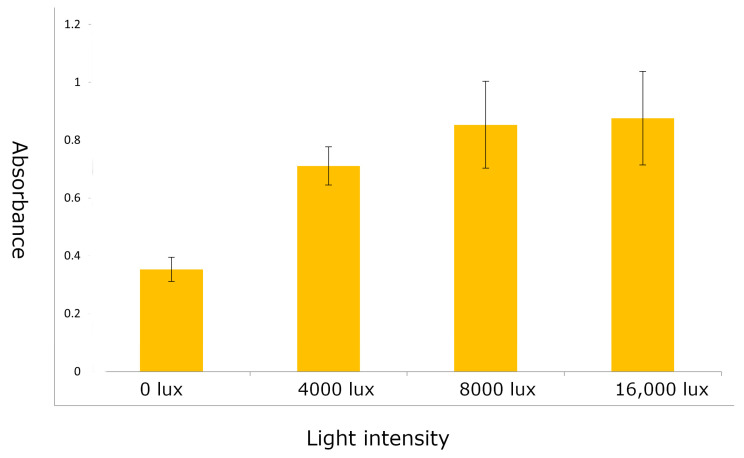
Anthocyanin accumulation in the embryos incubated on MS-RD medium exposed to different light intensities.

As light seems to be critical for anthocyanin expression, we tested if higher light intensities can enhance anthocyanin accumulation. Increasing light intensities showed a positive effect on anthocyanin accumulation in the embryos. Among different light intensities used (0, 4000, 8000, and 16,000 lux) tested, embryos exposed to 8000 lux and 16,000 lux showed the strongest anthocyanin expression compared with the embryos exposed to 4000 lux and embryos not exposed to light. (Figure 2). The 8000 lux light intensity was sufficient to induce good expression of anthocyanins.

**Figure 3:**
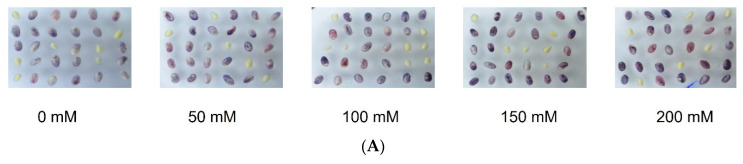

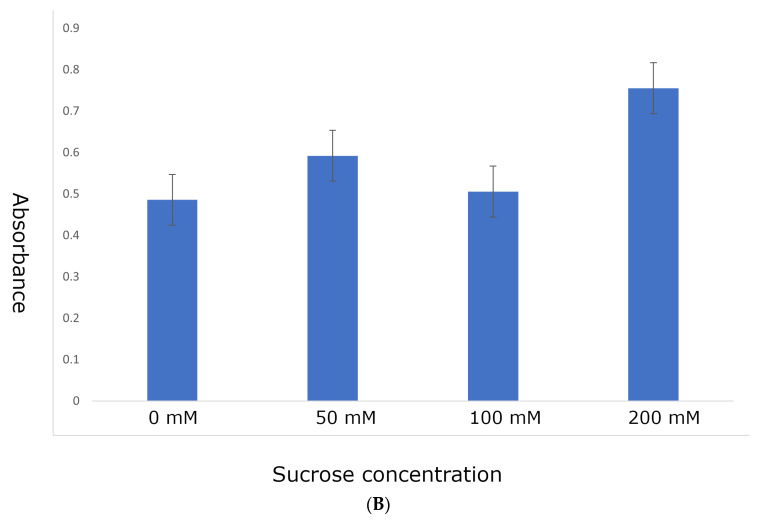
Effect of sucrose on anthocyanin accumulation. (**A**) Anthocyanin accumulation and (**B**) quantification of anthocyanins in embryos incubated on MS-RD medium supplemented with different concentrations of sucrose.

**Figure 4:**
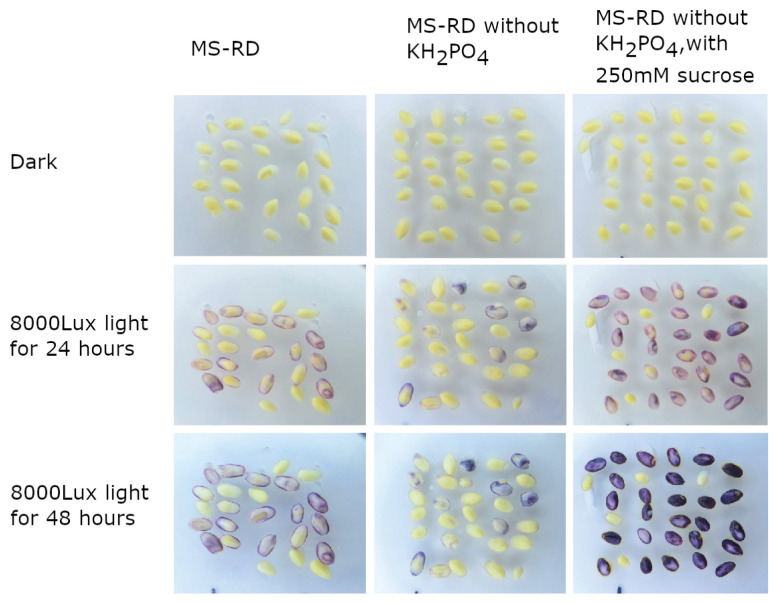
Effect of phosphorous deprivation on anthocyanin accumulation in embryos. Embryos were plated on different media for 24 and 48 h.

In the next experiment, we tested the effect of different concentrations of sucrose (0, 50, 100, 200 mM) on anthocyanin accumulation in embryos. Sucrose showed a positive effect on anthocyanin accumulation in embryos, especially at higher concentrations (Figure 3). However, the addition of sucrose without light did not affect anthocyanin accumulation (Figure 1 and Figure 4).

Further, we evaluated the effect of phosphorous deprivation in the medium and whether it further enhance anthocyanin accumulation. Embryos grown on normal MS-RD medium or phosphorous-deprived MS-RD medium did not show any anthocyanin expression when grown in the dark (Figure 4). Even when exposed to light for up to 48 h, embryos on phosphorous-deprived media did not show a significant accumulation of anthocyanins compared to normal MS-RD media under similar conditions. However, the addition of 250 mM sucrose to the phosphorous-deprived media resulted in a good accumulation of anthocyanins within 24 h.

**Figure 5:**
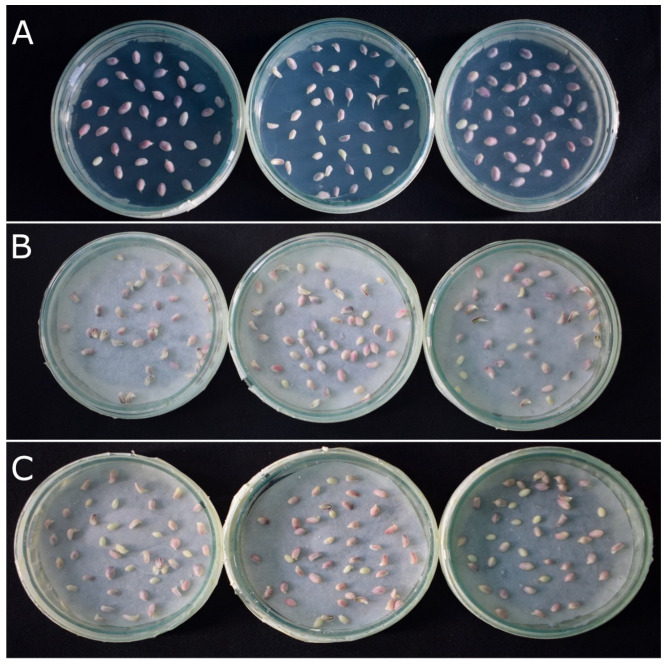
Anthocyanin accumulation in embryos on solid MS-RD, liquid MS-RD, and sucrose solutions incubated for 24 h under 8000 lux light intensity. (**A**) Embryos on solid MS-RD medium, (**B**) embryos on blotting paper saturated with liquid MS-RD medium, and (**C**) embryos on blotting paper saturated with 200 MM sucrose solution.

In addition, we also evaluated whether incubating embryos in sucrose solution was sufficient for anthocyanin accumulation. The results indicated that, similar to solid MS-RD and liquid MS-RD media, embryos could accumulate anthocyanins sufficiently in sucrose solution within 24 h when exposed to continuous light at 8000 lux (Figure 5). Together, a combination of 8000 lux light and 200 to 250 mM sucrose on blotting paper was sufficient for enhancing anthocyanin accumulation.

### 2.2. Verification of Accuracy of Haploid Embryo Identification Using R1-nj Marker

Plants obtained from putative haploid/diploid embryos were subjected to a gold standard test that is based on visual differences among haploids and diploids in plant characteristics such as seedling vigor, erectness of leaves, tassel fertility, etc. False discovery rate (FDR) varied from 8.3% to 43.4% with an average of 23.5% (Table 1). Three of the six populations showed a high FDR > 25%, indicating the presence of a significant proportion of false positives among haploids (true diploids falsely identified as haploids). False negative rate (FNR) varied from 0 to 8.3% with an average FNR of 1.2%. Four out of six populations resulted in an FNR of 2% or less, indicating that almost all diploid embryos identified based on the *R1-nj* marker were true diploids. In addition to the gold standard classification, we also used molecular markers for ascertaining true ploidy status in a subsection of putative haploids and diploids. Molecular markers indicated an average of 16.8% FDR and 2% FNR. Differences in FDR and FNR between gold standard tests and molecular tests can be attributed to the relatively small sample sizes used for molecular analysis. Two populations showed very high FDR (>40%) in the gold standard and molecular marker-based ploidy evaluation.

To study the accuracy of *R1-nj*-based haploid/diploid embryo identification in large numbers of embryos and diverse germplasm, we determined FDR in another 24 diverse tropical populations based on the gold standard test using plant traits (Table 2). The FDR values ranged from 4.9 to 82.1% with an average FDR of 19.4%. Five out of twenty-four populations showed high (>25%) FDR and only three populations showed less than 10% FDR, indicating significant false positives in the majority of the populations.

## 3. Discussion

Reducing the breeding cycle time is important for enhancing genetic gains and for the accelerated development of elite varieties with adaptation to climate change, and resistance to diseases and pests [22]. DH technology in maize can reduce breeding cycle time significantly compared with conventional pedigree and single-seed descent methods [22]. Currently, the process of deriving DH lines from a population using an in vivo haploid induction method takes two full crop cycles, including one cycle for haploid induction and one cycle for the production of seeds from haploid plants that were subjected to chromosomal doubling treatments, along with a significant time spent on identifying haploids. Typically, it takes five to six months per cycle from seed to seed in tropical maize germplasms; hence, it takes up to a year to derive DH lines. Even though it is quite fast to derive inbred lines via DH in two generations compared with six to eight generations in the recurrent selfing method, there is an opportunity to further reduce the time taken to develop DH lines by employing embryo rescue procedures. However, protocols that can be widely adapted for identifying haploid embryos and further doubling the chromosomes in the identified haploids are not yet published. Previously, patents published on the maize haploid embryo rescue process reported the use of transgenic marker systems for early (8–14 days after pollination) identification of haploid embryos [16,17]. These protocols may not be applicable in many countries in the Global South because of restrictions on the use of transgenics. Anthocyanin marker *R1-nj* is integrated into almost all the maternal haploid inducers [10], which can aid in haploid identification at the dry seed stage. Reports indicated that *R1-nj* expression on seeds starts 23 days after pollination at the silk attachment area [23] and is not expressed in embryos very early (8–12 days) [16,17]. However, it remains unexplored whether anthocyanin markers can be expressed at a slightly later stage and if they can be useful in haploid identification at the embryo stage.

In haploid induction crosses, the majority of the resulting embryos are diploid (~85–90%) when using the recent haploid inducers with a ~10–15% haploid induction rate. Hence, it would be best to eliminate the majority of diploid embryos that are of no use in DH line production before applying chromosomal doubling treatments. This will enable cost savings by reducing the quantity of chemicals used in chromosomal doubling, besides labor. Our experiments using tropicalized haploid inducers equipped with *R1-nj* markers indicated that the embryos extracted 18–30 days after pollination are ideal for embryo rescue in the tropical maize germplasm. However, when embryos were cultivated on MS-RD medium in the dark at 28 °C, anthocyanin accumulation was not generally observed. To achieve quick haploid embryo identification, we explored conditions that favor anthocyanin production in maize embryos resulting from haploid induction crosses.

Light is well-known to enhance anthocyanin production in plants [24,25,26,27,28]. Continuous white light can induce anthocyanin production in maize seedlings [29,30]. Light-induced expression of transcription factors that upregulate anthocyanin synthesis has been proposed to be responsible for the accumulation of anthocyanins by light in maize [17,29,31]. In our experiments, exposure to 8000–16,000 lux fluorescent white light greatly enhanced the expression of anthocyanins, confirming previous observations.

In addition to light, sugars stimulate anthocyanin accumulation in plant tissues. Among different sugars tested, sucrose specifically increases the anthocyanin content in Arabidopsis and upregulates the expression of anthocyanin biosynthetic pathway genes [32]. In maize, several sugars enhanced anthocyanin accumulation and upregulated anthocyanin regulatory genes [33]. Our experiments on maize embryos resulting from induction crosses also revealed that the addition of sucrose led to increased anthocyanin accumulation. Phosphorous deficiency greatly enhanced anthocyanin accumulation in in vitro cultures of coleoptiles of a white maize variety, while the same conditions did not enhance in a purple maize variety [34,35]. In our experiments, phosphorous deprivation under both dark and continuous light conditions (8000 lux) did not stimulate anthocyanin expression. However, embryos cultivated on a phosphorous-deprived media in the presence of light and sucrose showed anthocyanin accumulation, indicating that sucrose and light had a more positive effect than phosphorous deprivation on anthocyanin accumulation.

Together, exposure to continuous white light and the presence of sucrose was found to be sufficient to induce anthocyanins at levels that allow separation of haploid and diploid embryos. Our study also showed that haploid embryos can be separated from diploids with similar accuracy as that achieved with haploid seed sorting. In addition, our experiments also indicated that it is sufficient to incubate embryos in just sucrose solution on a blotting paper under continuous light for sufficient anthocyanin expression. This further simplifies the protocol for haploid/diploid embryo identification while reducing the incidence of bacterial or fungal contamination, which is more prevalent with the solid growing media as per our observations.

Two parameters are valuable in validating the accuracy of the haploid/diploid classification method, namely FDR and FNR [10]. FDR represents the proportion of false positives (true diploids) among putative haploids identified and FNR represents the proportion of true haploids among the putative diploids. Validation of the *R1-nj*-marker-based haploid embryo identification using a gold standard classification based on plant traits and molecular marker assays revealed high average FDR rates, while FNR was very low. The average FDR obtained when classifying haploids/diploid embryos using the *R1-nj* marker in this study was in a similar range to that reported for *R1-nj*-based haploid/diploid seeds in previous studies and varied significantly among the populations as indicated in earlier studies [9,36]. FDR is the most important criterion in haploid/diploid classification, as the presence of false positives results in investing additional resources in chromosomal doubling treatments and managing them in the greenhouse and in the field until they are properly identified. The presence of haploids among discarded diploids is a significantly lesser problem and can be easily addressed by increasing the number of embryos sorted for haploids, especially considering that the average FNR is 2%.

Even though the conditions reported can enhance anthocyanin expression, tropical maize germplasm and temperate flint germplasm contain dominant anthocyanin inhibitor genes (especially C1-I) at a high frequency [3,37]. Chaikam et al. (2016) [8] reported that ~40% of tropical maize populations showed segregation of *R1-nj* markers and ~4% populations showed complete inhibition of *R1-nj* markers. In populations with complete inhibition, it was impossible to use the *R1-nj* marker for haploid identification at the seed stage or embryo stage. In populations with a partial expression of the *R1-nj* marker, it was also impossible to separate true haploids from diploids due to the inhibition of *R1-nj* in some of the diploids. This results in a very high false-positive rate, as noticed in a few populations in this study. To mitigate the effects of complete or partial inhibition of *R1-nj* marker expression, we suggest two approaches. First, molecular markers reported earlier [8] can be used to identify populations with dominant anthocyanin color inhibitor genes before embryo rescue, thus saving valuable resources incurred in the process. Second, use of additional markers in combination with *R1-nj* will enable more effective identification of haploid embryos. The red root marker [9,38], high oil marker [10], and seedling traits were generally proposed to complement the *R1-nj* marker in identifying haploids at the seed/seedling stage. At the embryo stage, the high oil trait may not be useful unless it confers a visible difference in the embryo (e.g., embryo size); this needs to be further studied. The use of seedling traits also might not be very useful in embryo rescue cultures, as the seedlings derived from embryo cultures need to be grown for a longer duration in the greenhouse to detect these differences, which could be resource-intensive. Integration of the red root marker along with *R1-nj* could offer a more practical solution for identifying haploids from diploids in populations with complete anthocyanin color inhibition and reducing false positives in populations with partial inhibition, as red root marker can be expressed as soon as radicles emerge [9]. Together, the *R1-nj* marker could be used in some populations for the identification of haploid embryos, while in populations with anthocyanin inhibitor genes, it is not effective and the use of alternative markers needs to be explored.

## 4. Materials and Methods

### 4.1. Haploid Induction Crosses

Source populations used in the experiments described below were from the International maize and wheat improvement center’s (CIMMYT’s) tropical/sub-tropical breeding programs in Mexico and the mid-altitude breeding programs in Kenya and Zimbabwe. A haploid inducer hybrid, 2GTAIL006 × 2GTAIL009 [7], was used in all the haploid induction crosses, where both inducer parental lines were equipped with the *R1-nj* marker. Haploid induction nurseries were planted, managed, and crosses were carried out as described in a previous study [39].

### 4.2. Embryo Extraction

We initially attempted to extract embryos 13–15 days after pollination. However, the embryos were very small, fragile, and easily damaged, making them difficult to handle. In comparison, 18–30-day old embryos were found to be amenable for extraction; hence, embryos were extracted 18–30 days after pollination in all the experiments described in this manuscript. Embryos were extracted and plated as described earlier [40]. Harvested ears from the induction nursery were de-husked and surface-sterilized with 10% commercial bleach for 30 min. The ears were then rinsed three times with sterile distilled water. The crowns of the kernels were scraped off with a knife to expose the endosperm. Embryos were scooped out of the kennels using a sterile surgical blade. MS-RD medium (4.33 g/L Murashige and Skoog salts supplemented with 100 mg/L myo-inositol, 0.4 mg/L nicotinic acid, 0.2 mg/L thiamine-HCl, 20% sucrose, adjusted to pH 5.7–5.8 with 1 m KOH, solidified with 0.8% agar) [40] was used as a base embryo rescue culture medium.

### 4.3. Identifying Optimal Conditions for Anthocyanin Marker Expression

To determine if anthocyanin markers can aid in haploid/diploid embryo identification, embryos resulting from the induction crosses of two populations were plated on solid MS-RD medium and kept in the dark by wrapping them with aluminum foil or continuously exposed to light for 24, 48, 72, and 96 h in a growth chamber maintained at 28 °C. After the specified time period, the plates kept in the dark were unwrapped and visually inspected for anthocyanin marker expression in comparison to the embryos that were continuously exposed to the light.

To test the effect of light intensity on anthocyanin accumulation, embryos were plated on a solid MS-RD medium and were exposed to fluorescent light at different intensities (0, 4000, 8000, and 16,000 lux) for twenty-four hours on an incubator at 28 °C. To measure anthocyanin content, embryos exposed to different light intensities were individually weighed and placed in a microtube along with a metal bead; 200 µL of 95% ethanol and 100 µL of 0.1 M HCL were added. The embryos were crushed in a GenoGrinder at 1750 rpm for 30 s and centrifuged at 4000× *g* for 10 min. Anthocyanin was measured in the supernatant from the absorbance at 520 nm in a plate reader.

To test the effect of sucrose on anthocyanin accumulation, embryos were placed in MS-RD solid media with different concentrations of sucrose for 24 h in the dark. MS-RD medium contains 2% sucrose. For this experiment, 2% sucrose was omitted, and a specified concentration of sucrose (0, 50, 100, and 200 mM) was added. Anthocyanins were extracted and quantified as described above.

To test the effect of phosphorous deprivation on anthocyanin accumulation, MS-RD medium was prepared without the addition of a phosphorous source, namely, monopotassium phosphate (KH_2_PO_4_). Embryos were plated on this media with or without 200 mM sucrose and incubated for 24 and 48 h.

To test if a solid medium was required for anthocyanin accumulation, we grew the haploid embryos on MS-RD solid, MS-RD liquid medium, and a blotting paper saturated with 200 mM sucrose solution. All were exposed to 8000 lux light for 24 h.

### 4.4. Validation of R1-nj Marker-Based Haploid/Diploid Embryo Identification Was Based on a Gold Standard and Molecular Markers

Six different subtropical populations were crossed with haploid inducer hybrid 2GTAIL006 × 2GTAIL009 at Kiboko, Kenya, in the 2018 winter season. Embryos were extracted as described above and were placed on blotting papers saturated with 200 mM sucrose solution in Petri plates under inflorescent light at 8000 lux for 24 h in a growth chamber maintained at 28 °C. Haploids and diploid embryos were separated based on anthocyanin expression, with embryos showing purple or red color classified as diploids, while white or yellow embryos without purple/red coloration were classified as haploids. Both haploid and diploid embryos were planted in plastic seedling trays with peat moss in a polyhouse. The trays were watered with a solution containing macro and micronutrients. Leaf tissues were collected randomly from 16 haploids and 16 diploids from each population on the 12th day after planting the embryos in trays when the second leaf emerged. Plants were kept in the greenhouse for 25 days, after which both haploids and diploids were transplanted in the field. At the flowering stage, surviving plants were assessed for ploidy status by using a gold standard classification based on plant traits (less vigor, erect leaves, poor fertility of haploids, etc.) as described earlier [7,9,10,36] (Supplemental Figure S1). FDR and FNR were calculated as described in previous studies [8,9,10,36]. FDR is the proportion of diploid embryos misclassified as haploid embryos. FNR is the proportion of true haploid embryos misclassified as diploid embryos.

For molecular analysis, leaf tissues were sent to a genotyping service provider LGC Ltd., UK (lgcgroup.com) for DNA extraction and genotyping. A set of 97 single nucleotide polymorphism (SNP) markers from the CIMMYT quality control (QC) set were used for genotyping [41]. Samples were genotyped using LGC KASPar assays. Genotyping results indicated less than 1% missing data across all samples. Genotypic data were used to ascertain the ploidy status of each plant. If all the SNPs were homozygous, they were confirmed as haploids. If any SNP was present in a heterozygous condition, the samples were considered diploid. In the majority of diploids, a minimum of 20% heterozygosity was observed. FDR and FNR were calculated as described above.

To determine the accuracy of haploid/diploid embryo identification based on *R1-nj* marker expression in more diverse germplasm, 24 populations from CIMMYT-Kenya and CIMMYT-Zimbabwe breeding programs were induced for haploids between 2020 and 2022, and embryos were extracted 18–25 days after pollination. Embryos were incubated in sucrose solution for 24 h at specified light and temperature conditions in a growth chamber. Putative haploid embryos without anthocyanin coloration were separated, recovered in the greenhouse, and grown in the field. For accurate determination of ploidy, diploid plants were also grown from 25 embryos with purple color as controls for comparison (Supplemental Figure S1). Ploidy of the true haploids and false positives was established based on a gold standard classification based on seedling traits, and FNR was calculated.

## 5. Conclusions

This study attempted to address a critical limitation in adapting embryo rescue procedures in maize DH line production. The protocol proposed can assist in the effective identification of haploid embryos in 24 h using the *R1-nj* marker that is already incorporated in most of the haploid inducers commonly used in DH production pipelines. However, the presence of dominant anthocyanin color inhibitor genes may make the *R1-nj* marker ineffective in some maize populations, thereby necessitating the use of alternative approaches for identifying haploid embryos.

## Figures and Tables

**Table 1:** Validation of *R1-nj*-based haploid/diploid embryo identification using a gold standard classification based on plant traits and molecular markers.

Population	Gold Standard Classification			Molecular Marker Evaluation		
	N	FDR	FNR	N	FDR	FNR
(CKDHL0262/LH132)-B	276	30.0	0.0	30	0.0	0.0
(CKLMARS1C3S50264/PHW52)-B	180	43.4	3.2	32	75.0	20.0
(CKDHL0378/PHR03)-B	171	8.3	0.0	32	0.0	0.0
(CKDHL120390/LH198)-B	208	17.1	0.0	32	0.0	0.0
CKIR04003	144	28.6	6.3	32	25.0	7.7
CKDHL164288/CKDHL166062	203	12.3	3.0	32	0.0	11.1
	1182	23.5	1.2	190	16.8	2.0

N—Number of plants established from embryos and evaluated for ploidy level; FDR—False discovery rate; FNR—False negative rate.

**Table 2:** Determination of false discovery rates in diverse tropical populations using a gold standard classification based on plant traits.

Population	N	FDR
CZDHL150813/CKDHL164029	611	8.2
CZDHL150813/CKDHL163669	437	13.3
CZDHL151801/CKDHL163629	302	11.6
CKDHL165841/CKDHL166103	289	5.9
CZDHL151890/CKDHL163629	270	17.8
CKDHL150363/CKDHL152036	242	7.4
CZDHL153829/CKDHL163629	191	23.0
CKDHL0165/KS23-6	180	12.2
(CKL147/N21)-B	140	30.0
CZDHL155303/CZDHL154584	139	10.8
(CKL15117/PHAP1)-B	131	19.8
((CML536/CML463)DH173-B-B-1-B-B/PHW61)-B	128	35.9
(CKL15117/PHAP1)-B	82	4.9
(CKL1574/CL114162)-B	82	17.1
CZDHL153829/CKDHL164029	79	20.3
(DJL173833/PHAP1)-B	76	11.8
TZMI717/CML498	73	12.3
CKDHL153508/CKDHL164461	72	8.3
CML540LNT22/CKDHL0186LNT23	56	82.1
((CL114162/KS23-6)/CL114162)-B	53	43.4
(CKL141999/CL1211293)-B	48	8.3
CML572LNT23/CKL05017LNT22	40	20.0
((CML536/CML463)DH173-B-B-1-B-B/PHTV7)-B	33	12.1
TZMI717/NMCOMPOSITE2008-4-2-1-1-2-1	21	28.6
**Average FDR**		**19.4**

N—Number of plants established from embryos and evaluated for ploidy level; FDR—False discovery rate.

## Data Availability

The data used in the manuscript can be availed from the corresponding author upon reasonable request.

## Supplementary Materials

The following supporting information can be downloaded at: https://www.mdpi.com/article/10.3390/plants12122314/s1, Figure S1: Validation of true ploidy status of the putative haploids and putative diploids using a gold standard test based on differences in plant characteristics.
