# Supplementary figures and images for: Effectiveness of R1-nj Anthocyanin Marker in the Identification of In Vivo Induced Maize Haploid Embryos
Source: Plants (Basel). 2023 Jun 14;12(12):2314. doi: 10.3390/plants12122314 (PMC10302338; doi:10.3390/plants12122314)

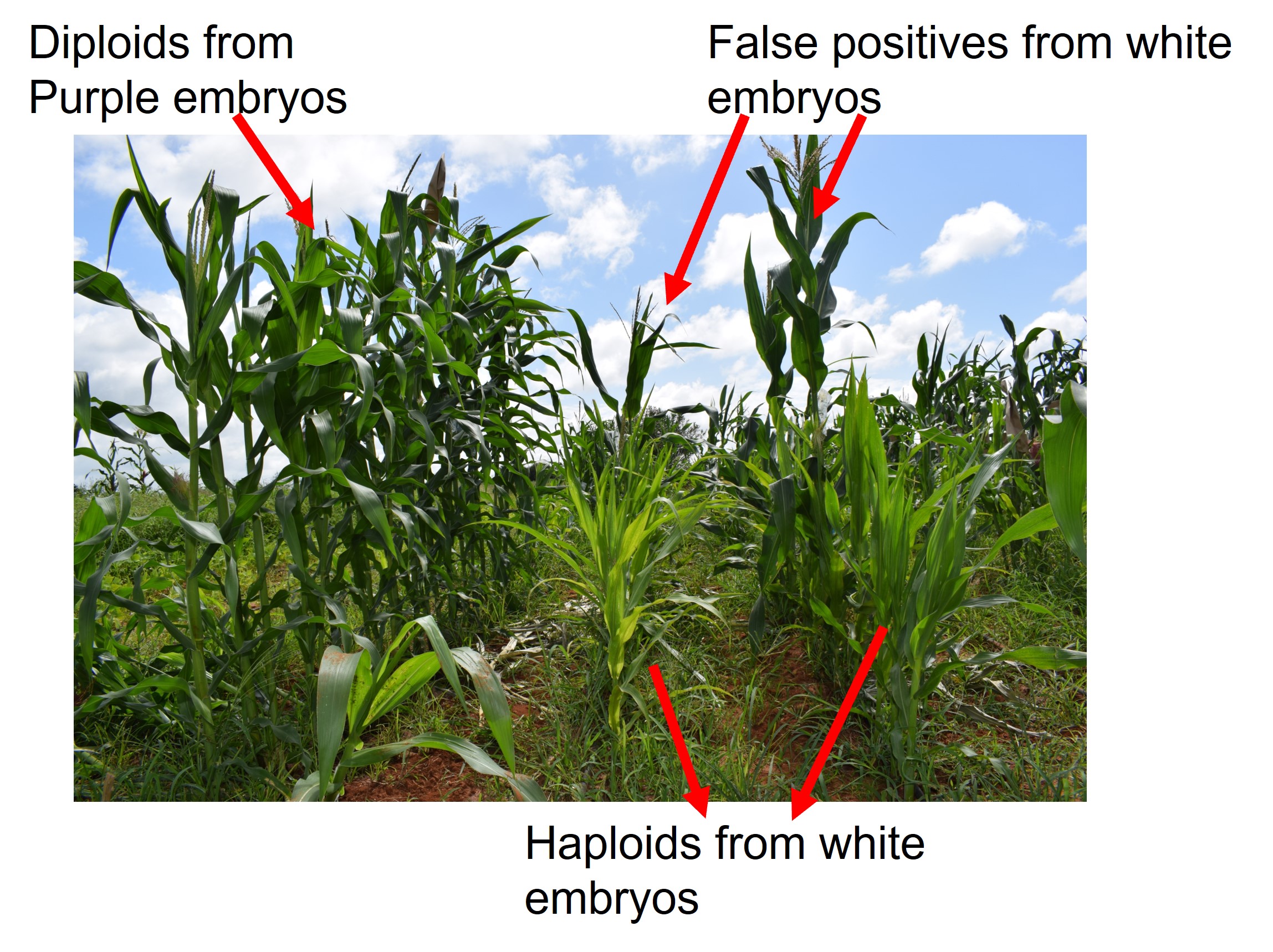

Supplement: Supplementary file 1 [file plants-12-02314-s001.zip › plants-2409692-supplementary.jpg]
